# p57 kip2 immunohistochemistry: ancillary technique in hydatidiform moles diagnosis

**DOI:** 10.1186/1753-6561-7-S2-P33

**Published:** 2013-04-04

**Authors:** Jessica Fernández, Rafael Cortés, Aleydah Salazar, Aníbal Pulido, Pablo Dabed, Victoria García

**Affiliations:** 1 Department of Obstetrics, Universitary Hospital of Caracas, Caracas, District Capital, Venezuela; 2 Institute of Pathology “José O´Daly”, Central University of Venezuela, Caracas, Venezuela

## Background

Gestational trophoblastic disease is a group of benign and malignant entities, where the hydatidiform mole is the most common, and can be divided into complete hydatidiform mole and partial hydatidiform mole. However, the histological criteria are still questionable and also, histologic evaluation alone is prone to great interobserver variability. In this way, inmunohistochemical analysis for p57 kip2 may be useful to aid in the diagnosis and classification of hydatidiform moles, with management and prognostic implications. p57 is the protein product of the paternally imprinted but maternally expressed gene CDKN1C. Because complete hydatidiform mole lack a maternal genomic component, an absence of p57 staining is specific for this pathology. This study looking for demonstrates the p57 kip2 immunohistochemistry as a technique useful in the differentiation between partial and complete hydatidiform mole.

## Patients and methods

From 2007 to 2011, our institute handled a total of 104 patients with gestational trophoblastic disease type complete and partial mole. This study did not consider patients with other forms of trophoblastic disease different to hydatidiform mole. The residues obtained after 104 curettages were analyzed by histological studies and 86 of them were subjected to determination of p57.

## Results

Of that total, 63.95% (55 patients) had a partial mole and the rest (31 patients, 36.04%) had a complete mole. There was divergence in the pre and post diagnostic immunohistochemistry in 12.79% of cases (11 patients).

## Conclusions

The p57 kip2 immunohistochemical technique should become a thorough job in diagnosing hydatidiform mole.

## Financial support

FAPESP and CAPES.

